# The Genetics of Reading Disability in an Often Excluded Sample: Novel Loci Suggested for Reading Disability in Rolandic Epilepsy

**DOI:** 10.1371/journal.pone.0040696

**Published:** 2012-07-18

**Authors:** Lisa J. Strug, Laura Addis, Theodore Chiang, Zeynep Baskurt, Weili Li, Tara Clarke, Huntley Hardison, Steven L. Kugler, David E. Mandelbaum, Edward J. Novotny, Steven M. Wolf, Deb K. Pal

**Affiliations:** 1 Child Health Evaluative Sciences, The Hospital for Sick Children, Toronto, Canada; 2 Dalla Lana School of Public Health, University of Toronto, Toronto, Canada; 3 Department of Clinical Neurosciences, Institute of Psychiatry, King’s College London, London, United Kingdom; 4 Department of Epidemiology, Mailman School of Public Health, Columbia University Medical Center, New York, New York, United States of America; 5 St. Christopher’s Hospital for Children, Philadelphia, Pennsylvania, United States of America; 6 Children’s Hospital of Philadelphia and University of Pennsylvania School of Medicine, Philadelphia, Pennsylvania, United States of America; 7 Hasbro Children’s Hospital and The Warren Alpert Medical School of Brown University, Providence, Rhode Island, United States of America; 8 Yale University Medical Center, New Haven, Connecticut, United States of America; 9 Beth Israel Medical Center, New York, New York, United States of America; 10 Department of Psychiatry, Columbia University Medical Center, New York, New York, United States of America; National Institute of Environmental Health Sciences, United States of America

## Abstract

**Background:**

Reading disability (RD) is a common neurodevelopmental disorder with genetic basis established in families segregating “pure” dyslexia. RD commonly occurs in neurodevelopmental disorders including Rolandic Epilepsy (RE), a complex genetic disorder. We performed genomewide linkage analysis of RD in RE families, testing the hypotheses that RD in RE families is genetically heterogenenous to pure dyslexia, and shares genetic influences with other sub-phenotypes of RE.

**Methods:**

We initially performed genome-wide linkage analysis using 1000 STR markers in 38 US families ascertained through a RE proband; most of these families were multiplex for RD. We analyzed the data by two-point and multipoint parametric LOD score methods. We then confirmed the linkage evidence in a second US dataset of 20 RE families. We also resequenced the *SEMA3C* gene at the 7q21 linkage locus in members of one multiplex RE/RD pedigree and the *DISC1* gene in affected pedigrees at the 1q42 locus.

**Results:**

In the discovery dataset there was suggestive evidence of linkage for RD to chromosome 7q21 (two-point LOD score 3.05, multipoint LOD 3.08) and at 1q42 (two-point LOD 2.87, multipoint LOD 3.03). Much of the linkage evidence at 7q21 derived from families of French-Canadian origin, whereas the linkage evidence at 1q42 was well distributed across all the families. There was little evidence for linkage at known dyslexia loci. Combining the discovery and confirmation datasets increased the evidence at 1q42 (two-point LOD = 3.49, multipoint HLOD = 4.70), but decreased evidence at 7q21 (two-point LOD = 2.28, multipoint HLOD  = 1.81), possibly because the replication sample did not have French Canadian representation.

**Discussion:**

Reading disability in rolandic epilepsy has a genetic basis and may be influenced by loci at 1q42 and, in some populations, at 7q21; there is little evidence of a role for known DYX loci discovered in “pure” dyslexia pedigrees. 1q42 and 7q21 are candidate novel dyslexia loci.

## Introduction

Reading disability (RD) is one of the most common developmental conditions in childhood with a prevalence ranging from 5–12% [1]. RD is defined as difficulty in reading and writing not attributable to general intellectual or sensory impairment or to a lack of exposure to an appropriate educational environment (ICD-10). RD may arise from a combination of environmental and genetic components, and there is evidence that the genetic susceptibility to RD is complex [2], [3], [4], [5], [6], [7], [8], [9], [10], [11], with over nine susceptibility loci (*DYX1-DYX9*) mapped [12]. Most genetic studies of RD are based on subjects in whom neurological comorbidities, like epilepsy, have been stringently excluded. Thus we do not know whether the genetic architecture of RD is the same in neurologically comorbid populations.

Epilepsy is often associated with neurodevelopmental disorders [13], and there is strong evidence that the association has a genetic basis in some instances. One common form of childhood epilepsy, Rolandic Epilepsy (RE), is a complex genetic disorder [14] whose electro-encephalographic signature (CentroTemporal Spikes, CTS) has been mapped to the *ELP4* gene at 11p13 [15]. RE is associated with language and academic impairments [16], including speech sound disorder (SSD) (odds ratio 2.5), and RD (OR 5.8). The odds of SSD in relatives of RE probands is 5.4 times that in the general population, and the odds of RD is 2.8 times the general population odds [17]. The strong proband and familial associations with RD and SSD, and the similarity in neurocognitive profiles between probands and siblings [18], suggest that these neurodevelopmental traits in RE are also genetically influenced. We subsequently tested the hypothesis of shared genetic influences for SSD and CTS, and have shown that the 11p13 locus is pleiotropic for SSD and CTS [19]. Other investigators have shown pleiotropic effects across neurodevelopmental phenotypes, for example between RD and SSD [2], [3]; RD and ADHD [20]; and RD and specific language impairment (SLI) [21].

The aim of this study was to map loci for RD in RE families and determine whether there is overlap with “pure” RD loci identified in non-comorbid samples. We asked two questions: (i) does reading disability in RE map to loci different from those so far reported in genetic studies of dyslexia in neurologically normal samples [2], [3], [4], and (ii) does reading disability share inheritance with CTS or SSD in RE families.

## Methods

### Ethics Statement

The IRBs of New York State Psychiatric Institute and all collaborating centers approved the study. All subjects gave written informed consent.

### Design

We employed a two-stage linkage design, the first stage was a genome-wide screen of 38 RE families; the second stage was a follow-up of linkage peaks and reported dyslexia loci in 20 later ascertained RE families. Where an obvious candidate existed, a gene under the linkage peak was resequenced.

### Ascertainment

Rolandic Epilepsy probands and their families were recruited from pediatric neurology centers in the US for a genetic linkage study in 2005–2007. Ascertainment was through the proband, with no requirement for other family members to be affected with epilepsy.

### Eligibility Criteria

Cases were enrolled if they met stringent eligibility criteria for RE, consisting of typical orofacial seizures, age of onset between 3–12 years, no previous epilepsy type, normal global developmental milestones, normal neurological examination, EEG with centrotemporal sharp waves and normal background, and neuroimaging that excluded an alternative structural, inflammatory or metabolic cause for the seizures. Board-certified experts in epileptology, neurophysiology, and neuroimaging centrally reviewed all of the probands’ charts, EEGs, and neuroimaging for eligibility prior to recruitment (see Acknowledgements). Questionable cases were discussed with an independent expert child neurologist specializing in epilepsy. Cases were not required to be comorbid with any neuropsychiatric disorder, and proband RD and SSD affectedness was unknown at time of ascertainment.

### Subjects

The first stage genome-wide linkage screen included 38 two or three generation RE families. In the second stage, we included an additional 20 two or three generation RE families. The number of genotyped individuals with RE, RD or both are listed for each family in Table S1.

### Phenotyping

A pediatric-trained physician (TC or DKP) interviewed the case families. Both parents were interviewed, either together or separately, and the proband and siblings were also interviewed when age appropriate. The investigator administered a 125-item questionnaire covering perinatal, developmental, medical, educational details, family history and detailed seizure semiology and treatment history. A pediatric neurologist, pediatric neuropsychologist, adult neuropsychologist, and pediatric speech pathologist jointly developed the questionnaire. Questions that were answered positively were followed up in detail by clinical interview to establish ICD-10 diagnoses and to distinguish from global learning disability. The questionnaire included 13 items addressing speech articulation disorder F80.0. A similar batch of questions was used in a high-risk study of phonological disorder (Tunick and Pennington, 2002). The questionnaire also contained nine items addressing the ICD-10 definitions of reading disorder F81.0. RD was thus identified by significant impairment in the development of reading skills not solely accounted for by mental age, sensory problems, mother tongue, or inadequate schooling. Operationally, we asked about difficulties in learning to read in the first year or two of elementary school, reading remediation, and repeating a grade. We also excluded, by clinical interview, hearing impairment, social and educational deprivation, and other factors that were inconsistent with the ICD-10 diagnosis of RD. We checked available school and psychologist’s reports for confirmation, and all were consistent with our findings.

A subset of 11 probands and 10 siblings underwent comprehensive neuropsychological evaluation, the details of which are reported elsewhere [18]. In brief, the results of detailed evaluation strongly supported the validity of our ICD-10 estimation of RD. As part of our battery, we used standard instruments to assess general intelligence: *Wechsler Abbreviated Scale of Intelligence*; academic achievement including spelling: *Woodcock-Johnson III* [*22*]; reading: *Gray Oral Reading Tests 4* [23], *Test of Word Reading Efficiency* [24]; receptive and expressive language: *Clinical Evaluation of Language Fundamentals, 4th Edition* [25], *Boston Naming Test*, *2nd Edition* [26]. All tested subjects had a full scale IQ within or above the normal range. Using a definition of impairment as a standard score one standard deviation below normative means in at least two subtests, we found that ICD-10 classifications had a 100% positive predictive value and 90% negative predictive value for reading impairment. At worst, our operational definitions slightly underestimated the actual prevalence of RD.

### Genotyping

Blood or saliva samples (Oragene, DNA Genotek, Canada) for DNA extraction were collected from probands and all potentially informative available family members. DNA was extracted using standard protocols (15). Individuals were classified as affected or not affected for Reading Disability according to clinical evaluation using operational ICD-10 definitions of reading disorder (F81.0) (who.int/classifications/en). In the first stage, a total of 194 individuals were genotyped at deCODE Genetics, Iceland using the deCODE 1000 marker single tandem repeat (STR) set, which has an average genome-wide resolution of 4 cM. In the second stage, a total of 145 individuals were typed for markers in the same STR set on chromosomes 1–3, 5–7, 11, 12 and 15–17. Twenty additional new markers were typed only in the replication sample. Amplified fragments were typed using ABI 3700 and ABI 3730 DNA analyzers with CEPH family DNA used as standards. Alleles were called automatically and checked for Mendelian consistency and Hardy-Weinberg equilibrium.

### Linkage Analysis

We analyzed the data by two-point and multipoint LOD score calculations using the Maximized Maximum LOD Score (MMLS) approach [27], an approximation to the MOD score method [28] which performs maximization over the full unknown trait model. The MMLS procedure specifies that one performs analysis twice, once under dominant and once under recessive disease locus assumptions [29], while keeping other unknown trait model parameters fixed. The consequent increase in type I error is equivalent to half a degree of freedom under a chi-squared distribution [27], and can be conservatively compensated for by increasing the LOD score critical value for significance by 0.3 LOD score units, ie to 3.3. We calculated two-point LOD scores under both dominant and recessive modes of inheritance [27], [30], [31], with a dominant gene frequency of 0.006, a recessive gene frequency at 0.1, a sporadic rate at 0.002, and penetrance of 0.50 [28], [32]. In regions providing significant evidence for linkage with RD, we then maximized over penetrance [27]. Marker allele frequencies were calculated from the dataset. We then followed up two-point results that provided LOD scores greater than 2.0 with multipoint analysis using Genehunter [33], again using the MMLS approach followed by penetrance maximization and computation of heterogeneity LOD scores. We also used multipoint analysis to follow up two-point LOD scores over 2.0 for different affectedness definitions to determine evidence for shared genetic factors, combining the traits: RD *or* CTS; and RD *or* SSD.

### Sequencing

We used bi-directional Sanger sequencing for mutation screening of the *SEMA3C* gene in a large 17-member pedigree, 10 affected with RD, of French-Canadian origin included in the original linkage analysis. Genomic DNA from 13 individuals was available for screening. Sanger sequencing was also used to screen the *DISC1* gene in 20 affected families with RE and RD. We designed primers to amplify all of the exons and alternatively spliced exons, as well as the splice sites, promoter and 5′ and 3′ UTRs. We sequenced DNA amplified by PCR using the ABI 3130 DNA Analyzer and interpreted sequence using the Invitrogen Vector NTI Advance Suite. See Table S2 for PCR primer Sequences.

## Results

### Genome-wide Linkage Analysis of Reading Disability

In two-point analysis of RD in the initial set of families, we observed a LOD score of 3.05 at marker D7S660 in chromosomal band 7q21. The LOD score maximized under a dominant mode of inheritance, with 60% penetrance, at a recombination fraction of 0.01. We also observed LOD scores above 2.0 on chromosomes 1, 2 and 6 (Table 1).

**Table 1:** Genome-wide LOD scores exceeding 2.0 in two-point MMLS linkage analysis for Reading Disability (RD) in original dataset; LOD scores for broader phenotypes (RD or Speech Sound Disorder (SSD) and RD or CentroTemporal Spikes (CTS)) at these loci also listed.

Chromosome	Marker (Flanking)	Max LOD RD	Max LOD RD or SSD	Max LOD RD or CTS
1q42-43	D1S1540 (D1S2709,D1S2850)	2.87 (1.90, −5.06)	1.46 (1.55 −1.62)	1.55 (0.76, −1.56)
2q14.2	D2S2254 (D2S363,D2S347)	2.10 (−0.98, −1.83)	1.24 (0.29, 0.31)	0.45 (0.74, 0.01)
2q14.2	D2S2116 (D2S286, D2S1777)	2.23 (0.12, 1.19)	2.20 (0.60, 1.93)	1.62 (0.77, 0.78)
6q25.2	D6S441 (D6S1687,D6S419)	2.11 (−0.42,1.06)	1.37 (0.04, 0.95)	0.48 (−0.29, 0.35)
7q21.11	D7S660 (D7S2443,D7S1528)	3.05 (2.73, −0.95)	1.75 (1.82, −1.14)	1.13 (1.46, 1.68)

The two-point LOD scores at all these markers decreased when the affectedness definition was extended to RD *or* SSD, and also for the affectedness definition RD *or* CTS (Table 1). Similarly, when we broadened the affectedness definition to RD *or* SSD *or* CTS at the 11p13 locus for CTS/SSD, the two-point LOD score at that locus decreased to -8.90, indicating no evidence of pleiotropy for RD with CTS/SSD at the 11p13 locus. We found little evidence for linkage to known dyslexia loci *DYX1*-*DYX8* in this dataset (Table 2).

**Table 2:** Maximum LOD scores at DYX loci in the original dataset ‘r’ indicates maximization of lod score under recessive model, ‘d’ under dominant model.

Locus	Chromosomal band	Closest marker	Max LOD	Model
DYX1	15q21	D15S659	0.008	30% r
DYX2	6p22.3-p21.3	D6S289	0.83	99% d
DYX3	2p16-p15	D2S337	0.54	30% d
DYX4	6q11.2-q12	D6S1557	0.35	30% d
DYX5	3p12-q13	D3S1276	0.55	85% d
DYX6	18p11.2	D18S453 (SPC/RDG)	0.64	99% r
DYX7	11p15.5	D11S4046	0.09	30% d
DYX8	1p36-p34	D1S2870	0.29	85% d

**Figure 1:**
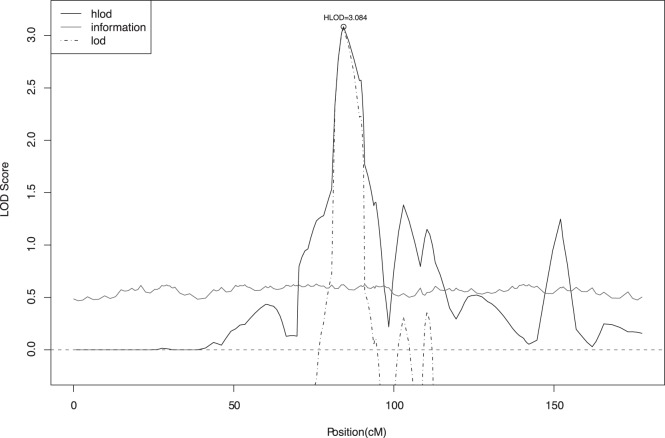
Multipoint analysis of Reading Disability on chromosome 7 in original dataset: maximum HLOD  = 3.08 at D7S660 under a dominant mode of inheritance with 50% penetrance. Black line shows the multipoint linkage evidence using a heterogeneity LOD score (HLOD); dotted black line shows the LOD score; and blue line the information content.

**Figure 2:**
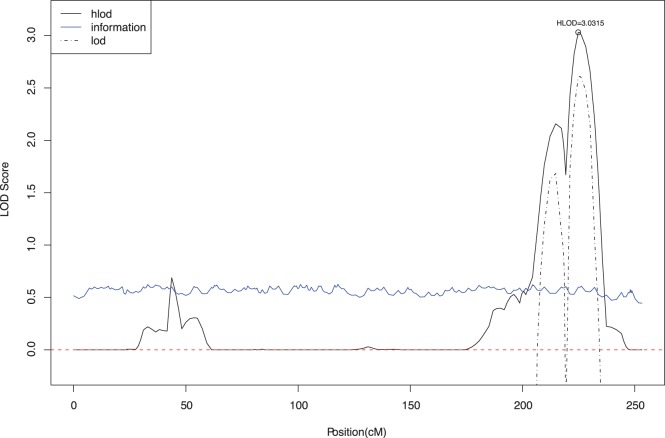
Multipoint analysis of Reading Disability on chromosome 1 in original dataset: maximum HLOD = 3.03 at D1S2833 under a dominant mode of inheritance with 60% penetrance. Black line shows the multipoint linkage evidence using a heterogeneity LOD score (HLOD); dotted black line shows the LOD score; and the blue line the information content.

We followed up our two-point results using multipoint analysis for the RD phenotype. Figure 1 shows the maximum multipoint LOD scores for RD on chromosome 7 under a dominant model with 50% penetrance. The largest multipoint LOD score was a HLOD of 3.08 at marker D7S660, the same marker where the maximum two-point score was found. The largest multipoint LOD score on chromosome 1 was a HLOD of 3.03 at marker D1S2833, maximizing under a dominant model with 60% penetrance (Figure 2). The LOD scores at chromosomes 2 and 6 were substantially smaller in multipoint compared to two-point analysis.

We repeated linkage analysis to RD in a second dataset of 20 RE families. In this replication set, we again found evidence of linkage to the 1q42 locus (LOD 1.28 at D1S2833, under a dominant 99% penetrance model). In a combined analysis of both datasets, the evidence for linkage of RD at 1q42 increased to LOD 3.55 at the same marker as in the original dataset, D1S1540, maximizing under a dominant, 70% penetrance trait model. In multipoint linkage analysis the maximum HLOD rose to 4.70 at D1S2833. However, at chromosome 7, the maximum LOD score in the second dataset was at D7S2409 (LOD 0.913, dominant, 99% penetrance), which was 10 cM distant from the linkage peak observed at D7S660 in the original dataset. In combined analysis, the evidence for linkage at D7S660 dropped to 2.28 (Table 3).

**Table 3:** Maximum LOD scores in the original, replication and combined datasets at chromosomes 1 and 7.

		Maximum two-point LOD			Max multipoint HLOD		
Chr	Marker	Original	Replication	Combined	Original	Replication	Combined
1q42-43	D1S2833	2.75	1.28	3.49	3.03	1.87	4.70
1q42-43	D1S1540	2.87	0.69	3.55	2.20	0.84	3.32
7q21.11	D7S2409	1.32	0.91	1.07	1.38	0.004	0.94
7q21.11	D7S2431	1.42	0.10	1.13	0.60	0.01	0.63
7q21.11	D7S660	3.05	0.134	2.28	3.08	0.00	1.81

**Figure 3:**
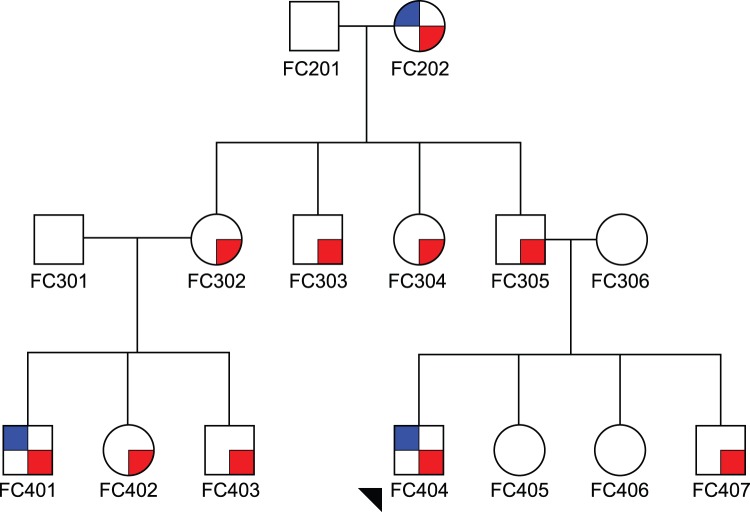
Pedigree of French-Canadian origin multiplex for RE (blue) and reading disability (red); arrow indicates proband.

Exploring the linkage data by family, sixteen families provided positive LOD scores at the 7q21 region, with the majority of the linkage evidence coming from eight French-Canadian families and three Hispanic families. Interestingly, there were no French-Canadian families in our replication dataset. One particular French-Canadian pedigree, (Figure 3) with 17 individuals (14 genotyped), 10 of which are RD affected, provided a multipoint LOD score of 2.10 at the same marker D7S660 under a dominant mode of inheritance with 99% penetrance. In fact, in a genome-wide analysis of this pedigree alone, D7S660 provided the maximum genome-wide multipoint LOD score. We therefore decided to conduct some resequencing of this pedigree at this locus.

### Resequencing SEMA3C

D7S660 sits in a gene-sparse region, except for the *SEMA3C* gene, which resides only 50,000 bp from the start of the microsatellite. Not only is this the closest gene to our linkage peak, but *SEMA3C* is an attractive candidate for further investigation. Semaphorins act as axonal growth cone guidance molecules, especially important in the developing fetal brain, and thus fit well with the theme emerging in the genetics of dyslexia and other neurodevelopmental disorders [34]. We sequenced the exons and alternatively spliced exons, exon-intron junctions, promoter, and 3′ and 5′ UTR of *SEMA3C* from all 13 individuals in the pedigree of French-Canadian origin (Figure 3) for whom we had DNA, (see Table S1 for primers).

No mutations or novel SNPs were found in *SEMA3C* in this pedigree. Eight known SNPs are present here although none segregated with affectedness status for RD. We did not find any evidence for microdeletions or microduplications in this gene. Therefore it is unlikely that *SEMA3C* coding or promoter mutations account for RD in this pedigree, and either intronic regions or other genes and ESTs under the linkage peak at 7q21 need to be investigated.

### Resequencing DISC1

The 1q42 locus contains approximately 25 genes in the 1-LOD interval around the linkage peak, and deletions at this locus have previously been associated with rolandic seizures and abnormal EEG [35]. The locus harbors an interesting candidate gene, *Disrupted in Schizophrenia 1* (*DISC1*) which is associated with many psychiatric and neurodevelopmental conditions [36], [37], [38]. We sequenced the coding, alternatively spliced and regulatory regions of *DISC1* in affected members of 20 families that were linked to the 1q42 locus.

We found 6 coding SNPs in the sequenced regions; however, none of them were novel, and all occurred at a frequency expected in a Caucasian population, indicating it is unlikely that *DISC1* coding mutations, microdeletions or microduplications, are causative for RD in RE families.

## Discussion

We have discovered evidence for possible novel dyslexia loci at 7q21 and 1q42 in families of subjects with rolandic epilepsy, a common type of epilepsy in which speech and language impairments strongly co-aggregate [17], [19]. The 7q21 and 1q42 loci have not previously been reported in the dyslexia genetics literature, suggesting that *DYX* loci discovered in relatively “pure” dyslexia samples may not be representative of genetic influences on RD found in samples comorbid for epilepsy. This provides further evidence for the genetic basis of comorbidity in common forms of epilepsy, following our report of pleiotropy between speech sound disorder and centrotemporal EEG spikes [19]. However, in contrast to findings of pleiotropy in non-comorbid RD pedigrees [2], [3], [20], we found no evidence for shared influences of these two loci for other neurodevelopmental traits associated with RE.

Approximately 55% of RE patients are co-morbid with RD [17], and many are affected by multiple neurodevelopmental comorbidities including SSD, attention deficit hyperactivity disorder (ADHD) and migraine [17], [18], [39]. The familial aggregation of these cognitive traits in relatives who may or may not have epilepsy or the subclinical EEG of CentroTemporal Spikes [17], [18], [39] enabled us to perform linkage analysis in pedigrees ascertained through rolandic epilepsy probands. This is in contrast to most published dyslexia genetic studies, which specifically exclude epilepsy and comorbid neurological conditions including SLI and ADHD. The two loci linked to RD in our RE sample have not been reported before, and we did not find confirmatory evidence of linkage at any of the DYX1-DYX8 loci. Our results suggest that the current list of dyslexia loci is not exhaustive, and that considering comorbid samples may increase the understanding of gene pathways, particularly the molecular basis for the observed overlap between conventionally distinct phenotypes such as epilepsy, ADHD, RD and SSD.

The most interesting candidate gene at the 1q42 locus is *DISC1*. *DISC1* has been linked and associated with schizophrenia and bipolar disorder in the general population [36], [37], and associated with autism and Asperger’s syndrome [38]. More specifically, *DISC1* mutations have been associated with deficits in sustained attention and memory in schizophrenia patients [40], [41], an observation that may be pertinent to the deficits in attention and working memory found in rolandic epilepsy [18], [42]. However, we did not identify any coding, splicing or regulatory mutations in *DISC1* from sequencing affected family members in this study, suggesting an alternative susceptibility gene, an intronic mutation, or other structural variations.

Our linkage results pointed to the possibility of a second, genetically heterogeneous RD locus enriched in French-Canadian pedigrees at 7q21. A chromosomal deletion at this locus (7q11.23q21.2) has previously been associated with microcephaly, ADHD, and epilepsy with bilateral centrotemporal EEG spikes [43]. The absence of coding and regulatory mutations in *SEMA3C* in one large multiplex French-Canadian pedigree suggests the need to consider other variation in or around this locus.

The genetic basis of dyslexia and speech sound disorder is firmly established in the neurodevelopmental genetics field, yet the occurrence of these common comorbidities among children with epilepsy has not previously been attributed to a genetic etiology. We recently showed pleiotropy at the 11p13 locus for both centrotemporal EEG spikes and speech dyspraxia [19] and this was the first evidence for genetic comorbidity in epilepsy. Here, we show that RD in RE also has a genetic basis and conclude that RD therefore does not result from either abnormal EEG or recurrent seizures. Since rolandic and most other common forms of epilepsy have a complex genetic basis and are often comorbid with RD, SSD, SLI, ADHD and/or migraine, it is reasonable to suggest that the etiological basis for some of these comorbidities in common epilepsies may also be genetic.

Pleiotropy has been demonstrated between SSD and RD in SSD families [2], [3], between RD and ADHD in RD families [20], and RD loci may be associated in comorbid individuals [21]. However, despite co-occurrence of RD and SSD in RE individuals and aggregation of both traits in RE families, neither familial aggregation nor linkage analysis suggests pleiotropy between RD and SSD in RE families [17]. These results suggest alternative genetic pathways may be involved in the etiology of these conditions in RE families compared to those in pure dyslexia families. We and others [21] propose that future genetic studies of RD, SSD, SLI and ADHD include collections of comorbid populations, because these traits in comorbid individuals may have genetic bases that are shared with or distinct from “pure” phenotypes. Moreover, the population prevalence and impact of comorbid individuals is likely highly significant. Delineation of genetic heterogeneity will improve statistical power to detect genetic influences. Studies including comorbid populations will contribute to a more comprehensive understanding of the genetic basis of common neurodevelopmental phenotypes.

## Supporting Information

Table S1 **Breakdown by pedigree of number affected with Rolandic Epilepsy, RE, Reading Disability, RD, or both, in original group 1:n and replication group 2:n.**
(DOC)

Table S2 **Forward and reverse primer sequences used in the PCR and subsequent Sanger sequencing of a.** ***SEMA3C*** **and b. DISC1** ***;*** **nomenclature from Ensembl.**
(DOC)
