# Supplementary material for: The Genetics of Reading Disability in an Often Excluded Sample: Novel Loci Suggested for Reading Disability in Rolandic Epilepsy
Source: PLoS One. 2012 Jul 18;7(7):e40696. doi: 10.1371/journal.pone.0040696 (PMC3399896; doi:10.1371/journal.pone.0040696)
Supplement: Table S1 — Breakdown by pedigree of number affected with Rolandic Epilepsy, RE, Reading Disability, RD, or both, in original group 1:n and replication group 2:n. (DOC) [file pone.0040696.s001.doc]

| Family | Number with RE only | Number with RD only | Number with RE & RD | Total number in pedigree |
| --- | --- | --- | --- | --- |
| 1:1 | 0 | 2 | 1 | 6 |
| 1:2 | 0 | 1 | 1 | 5 |
| 1:3 | 0 | 2 | 1 | 4 |
| 1:4 | 1 | 1 | 0 | 5 |
| 1:5 | 1 | 1 | 0 | 5 |
| 1:6 | 2 | 1 | 0 | 5 |
| 1:7 | 1 | 0 | 0 | 4 |
| 1:8 | 0 | 0 | 1 | 5 |
| 1:9 | 1 | 3 | 2 | 9 |
| 1:10 | 0 | 2 | 1 | 4 |
| 1:11 | 0 | 3 | 1 | 5 |
| 1:12 | 0 | 0 | 1 | 8 |
| 1:13 | 0 | 0 | 1 | 4 |
| 1:14 | 1 | 0 | 0 | 5 |
| 1:15 | 0 | 0 | 1 | 4 |
| 1:16 | 1 | 0 | 0 | 4 |
| 1:17 | 1 | 1 | 0 | 4 |
| 1:18 | 0 | 1 | 1 | 4 |
| 1:19 | 0 | 0 | 2 | 5 |
| 1:20 | 0 | 2 | 1 | 5 |
| 1:21 | 1 | 3 | 1 | 10 |
| 1:22 | 1 | 0 | 0 | 4 |
| 1:23 | 0 | 4 | 1 | 7 |
| 1:24 | 1 | 0 | 0 | 4 |
| 1:25 | 0 | 3 | 1 | 8 |
| 1:26 | 1 | 2 | 0 | 9 |
| 1:27 | 0 | 0 | 1 | 4 |
| 1:28 | 0 | 0 | 1 | 4 |
| 1:29 | 0 | 1 | 0 | 5 |
| 1:30 | 0 | 0 | 1 | 4 |
| 1:31 | 0 | 3 | 1 | 4 |
| 1:32 | 0 | 0 | 1 | 4 |
| 1:33 | 1 | 0 | 0 | 4 |
| 1:34 | 1 | 0 | 0 | 4 |
| 1:35 | 1 | 0 | 0 | 4 |
| 1:36 | 1 | 0 | 0 | 5 |
| 1:37 | 0 | 1 | 1 | 4 |
| 2:1 | 1 | 2 | 0 | 4 |
| 2:2 | 1 | 2 | 0 | 6 |
| 2:3 | 1 | 1 | 0 | 6 |
| 2:4 | 1 | 1 | 0 | 5 |
| 2:5 | 1 | 1 | 0 | 5 |
| 2:6 | 1 | 0 | 0 | 4 |
| 2:7 | 1 | 3 | 0 | 6 |
| 2:8 | 0 | 1 | 1 | 4 |
| 2:9 | 1 | 2 | 0 | 4 |
| 2:10 | 1 | 0 | 0 | 5 |
| 2:11 | 1 | 0 | 0 | 4 |
| 2:12 | 1 | 0 | 0 | 4 |
| 2:13 | 1 | 0 | 0 | 6 |
| 2:14 | 0 | 0 | 1 | 4 |
| 2:15 | 0 | 2 | 1 | 7 |
| 2:16 | 0 | 0 | 1 | 6 |
| 2:17 | 0 | 3 | 1 | 8 |
| 2:18 | 1 | 0 | 0 | 7 |
| 2:19 | 0 | 0 | 1 | 7 |
| 2:20 | 0 | 0 | 1 | 4 |

**Supplementary Table 1**. Breakdown by pedigree of number affected with Rolandic Epilepsy, RE, Reading Disability, RD, or both, in original group 1:n and replication group 2:n.
