# Supplementary material for: The Genetics of Reading Disability in an Often Excluded Sample: Novel Loci Suggested for Reading Disability in Rolandic Epilepsy
Source: PLoS One. 2012 Jul 18;7(7):e40696. doi: 10.1371/journal.pone.0040696 (PMC3399896; doi:10.1371/journal.pone.0040696)
Supplement: Table S2 — Forward and reverse primer sequences used in the PCR and subsequent Sanger sequencing of a. SEMA3C and b. DISC1 ; nomenclature from Ensembl. (DOC) [file pone.0040696.s002.doc]

| EXON | FORWARD | REVERSE |
| --- | --- | --- |
| 1 / 5’ UTR | gatatttacggccaggcaaa | ttctttcttcccggtcctct |
| 1 | gcgctgtaatcggacacc  gcgctgtaatcggacacc | ccaggcttgagcataccaat |
| 2 | gggtgttttgtctagattgctg | tttctgaatctggataaggaaga |
| 3 | cctcccaaagtgctgagatt | aaaagagacattgaaaaggtgga |
| 4 | ttgtgaaaggaaacactgttgg | catttttggcttccctcaaa |
| 5 | tgtcacctcattgttacttatcataca | ctctaaaatacagtttcaaaaatcctt |
| 6 | aagcaaccaactagaaagcatatt | tcatccacacaaccatcaaga |
| 7 | gctggacaatgcaaaccaat | aaccattttccctagttgttagga |
| 8 | aggcatctgcctgtggtc | aaaagcacggagataaacca |
| 9 | ttcatcctccaagcaactagag | ggaaagtacatcttcttttgagacc |
| 10 | gggtaaatttttcaaaacaaagaa | ttaaaagttgcagagaagaatgc |
| 11 | tcagcttttcacatgtgttcg | aaaaccccacagatattcattacc |
| 12 | ctggagcttctaagtgatttcaa | catggcccttacctactacct |
| 13 | tggtccaagtcagtagtcctatgt | cctcccaagtggctgtaaga |
| 14 | agaaaagtgatttcttcttgagattt | aaattaaaggtgactttgcggta |
| 15 | atgaatccaagggtttgtgg | aatgtcatttaaggaaacagcaa |
| 16 | gcaataaaaagggtgcaaagtt | tgacaatgtttccccttactga |
| 17 | ctggctgcgatagttgttca | ctcgctgagggacagtgatt |
| 18 | ttcccaaatctgtgaaagttga | aaagcacaagtttctttgctca |
| 3’ UTR 1 | tgagaggggactatggcaag | caactttaaatgctgtcttcctttt |
| 3’ UTR 2 | aacaagttccaaatccacagc | tgaagttgaaaactagccatatttgt |
| 3’ UTR 3 | ggtgtgaaatcatcaaaaagaaaa | atgatatttggattaggtacatggt |
| 3’ UTR 4 | gacattttcatgcttccaggt | tcagtttgggcttatgtgaaa |
| VARIANT 2 EXON 9 | tgcctcttgagtatgtagggaaa | gccatgttaggaaagtaacagca |
| VARIANT 3 EXON 1 | gctaatgtaacccaaggagacg | tcaatgagagaacgttgaagg |
| VARIANT 5 EXON 3 | caaaacaaaattacattcaattcca | caatgggtttaaagctgatgc |
| VARIANT 6 EXON 1 | tgtgttgttagtttgcgatcag | ccccttggttctgttgactt |

Supplementary Table S2a. Forward and reverse primer sequences used in the PCR and subsequent Sanger sequencing of *SEMA3C.*

| EXON | FORWARD | REVERSE |
| --- | --- | --- |
| 1, 1a and 5’ UTR | ggactcgctgaggagaagaaa | cacctcgaaaggggttgtta |
| 2 and 2a first half | ttctccagatgcagttccag | ggctgcagctgttgctact |
| 2 and 2a second half | agcaggctctctgccatc | cgtctgcccagtgctaatct |
| 3 | tcacaaaaatgtttgcttgaat | gatggaaagaaaattgggaca |
| 4 | agtgattctcctgcctcagc | tgttgaatgttcccaacacaa |
| 5 | ccggggttatctattttgcat | tgaggggaaaatggtgaca |
| 6 | tgaggatttcagcttctgcat | gcaagaccctgtctcaaagaa |
| 7 | tcagagcagtttgccatgag | ccaatgaacaggtcaaagagg |
| 8 and 8a | tggaaggttcactttttgcag | tttgcagaagccaggtatcc |
| 9 and 9a | atctctgacctggctgttcc | cacgatgtgctggtagctgt |
| 10 and 10a | cttccatgtgtgtggatgct | caatatcttgccggggaac |
| 11 and 11a | cctcaatcctttggctttga | ccagcctttttcatcgattt |
| 12 and 12a,b,c,d | tgaccagctgacttttagcc | atgccaaaagttgggttttt |
| 13 | tctgtgtccacggcactaac | ctgaggcatgaaaaactaccc |
| 14 and 3’UTR | ctcacacgctcttcgatcc | attttcgttcgagcagaagg |
| 3’ UTR 2 | ttgggaatgtcttccacagg | tgcactgctttcctaaatgc |
| Variant exon 004 and 4a | cgaacaagtgtgtccagcat | tcatgatctacgctgtgtgg |
| Variant exon 006 | ttacaaggctccaggcactt | tgtctcttcaatgccctttca |
| Variant exon 205 | gcacactttgattggtcagc | gcaaaaattaggttaacaggatca |
| Variant exon 202 | gagatggagtctcactctgtcg | tccttacctagaatttcttatttttca |

# Supplementary Table S2b. Forward and reverse primer sequences used in the PCR and subsequent Sanger sequencing of *DISC1*. Nomenclature form Ensembl.
